# 2-(2,2,2-Trifluoro­eth­yl)isoindoline-1,3-dione

**DOI:** 10.1107/S1600536810020222

**Published:** 2010-06-23

**Authors:** Xian-Shu Fu, Xiao-Ping Yu, Wei-Min Wang, Fang Lin

**Affiliations:** a College of Life Sciences, China Jiliang University, Hangzhou 310018, People’s Republic of China

## Abstract

In the title compound, C_10_H_6_F_3_NO_2_, the isoindole ring system is planar, the maximum atomic deviation being 0.012 (2) Å. The C—C bond of the trifluoro­ethyl group is twisted with respect to the isoindole ring by a dihedral angle of 62.58 (17)°. Weak inter­molecular C—H⋯O and C—H⋯F hydrogen bonding is present in the crystal structure.

## Related literature

The title compound is a key inter­mediate in the synthesis of organic electro-luminescent materials, see: Han & Kay (2005 ▶). For the synthesis, see: Valkonen *et al.* (2007 ▶); Barchin *et al.* (2002 ▶). For a related structure, see: Valkonen *et al.* (2007 ▶).
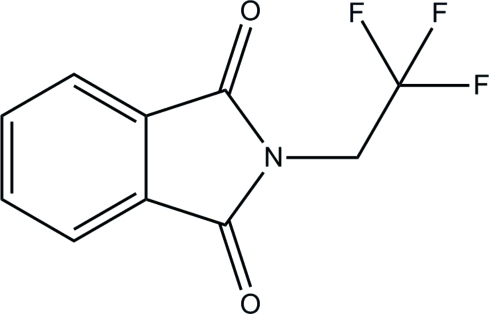


## Experimental

### 

#### Crystal data

C_10_H_6_F_3_NO_2_
*M* *_r_* = 229.16
Monoclinic, 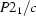

*a* = 5.047 (1) Å
*b* = 9.5370 (19) Å
*c* = 19.051 (4) Å
β = 95.20 (3)°
*V* = 913.2 (3) Å^3^
*Z* = 4
Mo *K*α radiation
μ = 0.16 mm^−1^
*T* = 113 K
0.26 × 0.18 × 0.14 mm

#### Data collection

Rigaku Saturn CCD area-detector diffractometer
Absorption correction: multi-scan (*CrystalClear*; Rigaku, 2005 ▶) *T*_min_ = 0.960, *T*_max_ = 0.978
7732 measured reflections
1608 independent reflections
1009 reflections with *I* > 2σ(*I*)
*R*_int_ = 0.083

#### Refinement

*R*[*F* ^2^ > 2σ(*F* ^2^)] = 0.055
*wR*(*F* ^2^) = 0.126
*S* = 0.97
1608 reflections
146 parameters
H-atom parameters constrained
Δρ_max_ = 0.43 e Å^−3^
Δρ_min_ = −0.50 e Å^−3^

### 

Data collection: *CrystalClear* (Rigaku, 2005 ▶); cell refinement: *CrystalClear*; data reduction: *CrystalClear*; program(s) used to solve structure: *SHELXS97* (Sheldrick, 2008 ▶); program(s) used to refine structure: *SHELXL97* (Sheldrick, 2008 ▶); molecular graphics: *SHELXTL* (Sheldrick, 2008 ▶); software used to prepare material for publication: *CrystalStructure* (Rigaku, 2005 ▶).

## Supplementary Material

Crystal structure: contains datablocks I, global. DOI: 10.1107/S1600536810020222/sj5001sup1.cif

Structure factors: contains datablocks I. DOI: 10.1107/S1600536810020222/sj5001Isup2.hkl

Additional supplementary materials:  crystallographic information; 3D view; checkCIF report

## Figures and Tables

**Table 1:** Hydrogen-bond geometry (Å, °)

*D*—H⋯*A*	*D*—H	H⋯*A*	*D*⋯*A*	*D*—H⋯*A*
C6—H6⋯O2^i^	0.95	2.52	3.462 (3)	174
C4—H4⋯O1^ii^	0.95	2.60	3.229 (3)	124
C9—H9*B*⋯O2^iii^	0.99	2.54	3.311 (3)	135
C3—H3⋯F3^ii^	0.95	2.62	3.556 (3)	168
C3—H3⋯O1^ii^	0.95	2.66	3.247 (3)	120

Symmetry codes: (i) 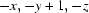; (ii) 
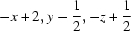
; (iii) 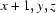.

**Fig. 1.**
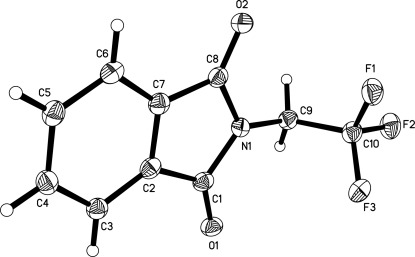
The molecule of I showing the atom-labelling scheme. Displacement ellipsoids are drawn at the 30% probability level.

**Fig. 2.**
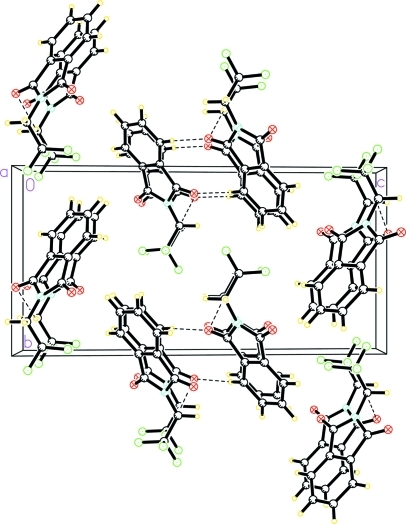
Crystal packing of I viewed down the a axis with hydrogen bonds drawn as dashed lines.

## supplementary crystallographic information

### Comment

The title compound, I, is a key intermediate in the synthesis of organic electro-luminescent materials. The emission of light by organic molecules exposed to an electric field has been widely investigated in both an academic and industrial context. (Han & Kay, 2005).

The molecular structure of the title compound is illustrated in Fig. 1. The isoindole ring system is planar, the maximum atomic deviation being 0.012 (2) Å (for the N1 atom). The C9—C10 bond of the trifluoroethyl group is twisted with respect to the isoindole ring by a dihedral angle of 62.58 (17)°, which is similar to the angle 60.3 (5)° found in the related compound 2-(2-iodoethyl)isoindole-1,3-dione (Valkonen *et al.* 2007). Weak intermolecular  C—H···O and and C—H···F hydrogen bonding is present in the crystal structure, Table 1, Fig. 2.

### Experimental

An acetic acid solution of phthalic anhydride (14.8 g, 100 mmol) and 2,2,2-trifluoroethylamine (7.99 ml, 100 mmol) was refluxed overnight, and then filtered. The crude product was recrystallized from ethyl acetate.

### Refinement

H atoms were positioned geometrically and refined as riding with C—H = 0.95 or 0.99 Å, and U_iso_(H) = 1.2U_eq_(C).

### Crystal data

**Table:**

C_10_H_6_F_3_NO_2_	*F*(000) = 464
*M**_r_* = 229.16	*D*_x_ = 1.667 Mg m^−^^3^
Monoclinic, *P*2_1_/*c*	Melting point: 400 K
Hall symbol: -P 2ybc	Mo *K*α radiation, λ = 0.71075 Å
*a* = 5.047 (1) Å	Cell parameters from 2545 reflections
*b* = 9.5370 (19) Å	θ = 2.1–28.0°
*c* = 19.051 (4) Å	µ = 0.16 mm^−^^1^
β = 95.20 (3)°	*T* = 113 K
*V* = 913.2 (3) Å^3^	Prism, colorless
*Z* = 4	0.26 × 0.18 × 0.14 mm

### Data collection

**Table:**

Rigaku Saturn CCD area-detector diffractometer	1608 independent reflections
Radiation source: rotating anode	1009 reflections with *I* > 2σ(*I*)
multilayer	*R*_int_ = 0.083
ω and φ scans	θ_max_ = 25.2°, θ_min_ = 2.2°
Absorption correction: multi-scan (*CrystalClear*; Rigaku, 2005)	*h* = −6→5
*T*_min_ = 0.960, *T*_max_ = 0.978	*k* = −11→11
7732 measured reflections	*l* = −22→22

### Refinement

**Table:**

Refinement on *F*^2^	Secondary atom site location: difference Fourier map
Least-squares matrix: full	Hydrogen site location: inferred from neighbouring sites
*R*[*F*^2^ > 2σ(*F*^2^)] = 0.055	H-atom parameters constrained
*wR*(*F*^2^) = 0.126	*w* = 1/[σ^2^(*F*_o_^2^) + (0.0618*P*)^2^] where *P* = (*F*_o_^2^ + 2*F*_c_^2^)/3
*S* = 0.97	(Δ/σ)_max_ < 0.001
1608 reflections	Δρ_max_ = 0.43 e Å^−^^3^
146 parameters	Δρ_min_ = −0.50 e Å^−^^3^
0 restraints	Extinction correction: *SHELXL97* (Sheldrick, 2008), Fc^*^=kFc[1+0.001xFc^2^λ^3^/sin(2θ)]^-1/4^
Primary atom site location: structure-invariant direct methods	Extinction coefficient: 0.301 (15)

### Special details

**Table:**

Geometry. All esds (except the esd in the dihedral angle between two l.s. planes) are estimated using the full covariance matrix. The cell esds are taken into account individually in the estimation of esds in distances, angles and torsion angles; correlations between esds in cell parameters are only used when they are defined by crystal symmetry. An approximate (isotropic) treatment of cell esds is used for estimating esds involving l.s. planes.
Refinement. Refinement of *F*^2^ against ALL reflections. The weighted *R*-factor wR and goodness of fit *S* are based on *F*^2^, conventional *R*-factors *R* are based on *F*, with *F* set to zero for negative *F*^2^. The threshold expression of *F*^2^ > σ(*F*^2^) is used only for calculating *R*-factors(gt) etc. and is not relevant to the choice of reflections for refinement. *R*-factors based on *F*^2^ are statistically about twice as large as those based on *F*, and *R*- factors based on ALL data will be even larger.

### Fractional atomic coordinates and isotropic or equivalent isotropic displacement parameters (Å^2^)

**Table:**

	*x*	*y*	*z*	*U*_iso_*/*U*_eq_	
F1	0.3850 (3)	0.94405 (16)	0.09725 (9)	0.0441 (5)	
F2	0.7282 (3)	1.04965 (15)	0.06326 (9)	0.0434 (6)	
F3	0.7509 (3)	0.94503 (15)	0.16352 (8)	0.0424 (5)	
O1	1.0018 (3)	0.65493 (18)	0.17852 (9)	0.0311 (5)	
O2	0.2715 (3)	0.63673 (19)	0.01945 (9)	0.0327 (6)	
N1	0.6488 (4)	0.6796 (2)	0.09384 (11)	0.0258 (6)	
C1	0.7960 (5)	0.6112 (3)	0.14966 (14)	0.0259 (7)	
C2	0.6475 (5)	0.4813 (3)	0.16200 (13)	0.0249 (7)	
C3	0.7001 (5)	0.3752 (3)	0.21083 (14)	0.0288 (7)	
H3	0.8508	0.3792	0.2445	0.035*	
C4	0.5253 (5)	0.2632 (3)	0.20895 (14)	0.0306 (7)	
H4	0.5581	0.1884	0.2416	0.037*	
C5	0.3044 (5)	0.2576 (3)	0.16073 (15)	0.0316 (7)	
H5	0.1870	0.1798	0.1611	0.038*	
C6	0.2508 (5)	0.3642 (3)	0.11158 (14)	0.0289 (7)	
H6	0.0992	0.3609	0.0782	0.035*	
C7	0.4270 (5)	0.4746 (3)	0.11336 (13)	0.0258 (7)	
C8	0.4261 (5)	0.6019 (3)	0.06811 (14)	0.0264 (7)	
C9	0.7342 (5)	0.8059 (3)	0.06062 (14)	0.0296 (7)	
H9A	0.6593	0.8074	0.0108	0.036*	
H9B	0.9306	0.8055	0.0613	0.036*	
C10	0.6488 (6)	0.9351 (3)	0.09660 (15)	0.0326 (7)	

### Atomic displacement parameters (Å^2^)

**Table:**

	*U*^11^	*U*^22^	*U*^33^	*U*^12^	*U*^13^	*U*^23^
F1	0.0356 (11)	0.0349 (10)	0.0619 (13)	0.0086 (8)	0.0059 (8)	−0.0084 (8)
F2	0.0571 (13)	0.0238 (10)	0.0492 (12)	−0.0014 (8)	0.0039 (9)	0.0066 (7)
F3	0.0596 (13)	0.0298 (10)	0.0362 (11)	0.0038 (8)	−0.0051 (9)	−0.0039 (7)
O1	0.0285 (12)	0.0274 (11)	0.0361 (11)	0.0003 (9)	−0.0034 (9)	−0.0039 (8)
O2	0.0298 (12)	0.0350 (12)	0.0321 (12)	0.0042 (8)	−0.0040 (9)	0.0001 (9)
N1	0.0227 (13)	0.0211 (12)	0.0329 (13)	0.0022 (10)	−0.0022 (10)	0.0009 (10)
C1	0.0249 (16)	0.0237 (15)	0.0288 (15)	0.0043 (13)	0.0003 (12)	−0.0047 (11)
C2	0.0213 (15)	0.0247 (15)	0.0287 (15)	0.0059 (12)	0.0015 (12)	−0.0037 (11)
C3	0.0277 (17)	0.0281 (16)	0.0305 (16)	0.0069 (13)	0.0012 (12)	−0.0020 (12)
C4	0.0331 (18)	0.0248 (16)	0.0347 (17)	0.0052 (13)	0.0081 (13)	0.0022 (12)
C5	0.0283 (17)	0.0250 (16)	0.0423 (18)	0.0009 (13)	0.0073 (14)	−0.0057 (12)
C6	0.0261 (17)	0.0277 (16)	0.0327 (16)	0.0018 (13)	0.0018 (12)	−0.0072 (12)
C7	0.0245 (16)	0.0232 (15)	0.0303 (16)	0.0038 (12)	0.0053 (12)	−0.0040 (12)
C8	0.0230 (16)	0.0255 (15)	0.0305 (16)	0.0057 (12)	0.0011 (12)	−0.0051 (12)
C9	0.0302 (16)	0.0237 (15)	0.0347 (17)	0.0021 (13)	0.0018 (12)	0.0027 (12)
C10	0.0340 (19)	0.0238 (16)	0.0389 (18)	0.0009 (14)	−0.0022 (13)	0.0032 (12)

### Geometric parameters (Å, °)

**Table:**

F1—C10	1.335 (3)	C3—H3	0.9500
F2—C10	1.343 (3)	C4—C5	1.380 (3)
F3—C10	1.334 (3)	C4—H4	0.9500
O1—C1	1.205 (3)	C5—C6	1.392 (4)
O2—C8	1.203 (3)	C5—H5	0.9500
N1—C8	1.398 (3)	C6—C7	1.376 (3)
N1—C1	1.402 (3)	C6—H6	0.9500
N1—C9	1.445 (3)	C7—C8	1.489 (4)
C1—C2	1.478 (4)	C9—C10	1.492 (4)
C2—C3	1.384 (3)	C9—H9A	0.9900
C2—C7	1.383 (3)	C9—H9B	0.9900
C3—C4	1.384 (4)		
			
C8—N1—C1	111.9 (2)	C5—C6—H6	121.4
C8—N1—C9	123.4 (2)	C6—C7—C2	122.0 (2)
C1—N1—C9	124.2 (2)	C6—C7—C8	129.9 (2)
O1—C1—N1	124.1 (2)	C2—C7—C8	108.1 (2)
O1—C1—C2	130.3 (2)	O2—C8—N1	124.6 (2)
N1—C1—C2	105.6 (2)	O2—C8—C7	129.7 (2)
C3—C2—C7	120.7 (2)	N1—C8—C7	105.6 (2)
C3—C2—C1	130.6 (2)	N1—C9—C10	112.2 (2)
C7—C2—C1	108.7 (2)	N1—C9—H9A	109.2
C2—C3—C4	117.7 (2)	C10—C9—H9A	109.2
C2—C3—H3	121.2	N1—C9—H9B	109.2
C4—C3—H3	121.2	C10—C9—H9B	109.2
C5—C4—C3	121.4 (2)	H9A—C9—H9B	107.9
C5—C4—H4	119.3	F3—C10—F1	106.6 (2)
C3—C4—H4	119.3	F3—C10—F2	106.8 (2)
C4—C5—C6	121.1 (2)	F1—C10—F2	107.0 (2)
C4—C5—H5	119.5	F3—C10—C9	113.3 (2)
C6—C5—H5	119.5	F1—C10—C9	112.8 (2)
C7—C6—C5	117.1 (2)	F2—C10—C9	110.1 (2)
C7—C6—H6	121.4		
			
C8—N1—C1—O1	176.9 (2)	C1—C2—C7—C6	179.5 (2)
C9—N1—C1—O1	4.5 (4)	C3—C2—C7—C8	−179.4 (2)
C8—N1—C1—C2	−2.3 (3)	C1—C2—C7—C8	−0.4 (3)
C9—N1—C1—C2	−174.7 (2)	C1—N1—C8—O2	−178.4 (2)
O1—C1—C2—C3	1.4 (5)	C9—N1—C8—O2	−6.0 (4)
N1—C1—C2—C3	−179.5 (3)	C1—N1—C8—C7	2.0 (3)
O1—C1—C2—C7	−177.6 (3)	C9—N1—C8—C7	174.5 (2)
N1—C1—C2—C7	1.6 (3)	C6—C7—C8—O2	−0.4 (5)
C7—C2—C3—C4	0.1 (4)	C2—C7—C8—O2	179.5 (3)
C1—C2—C3—C4	−178.7 (3)	C6—C7—C8—N1	179.1 (3)
C2—C3—C4—C5	−0.7 (4)	C2—C7—C8—N1	−1.0 (3)
C3—C4—C5—C6	0.7 (4)	C8—N1—C9—C10	100.0 (3)
C4—C5—C6—C7	−0.1 (4)	C1—N1—C9—C10	−88.4 (3)
C5—C6—C7—C2	−0.5 (4)	N1—C9—C10—F3	61.7 (3)
C5—C6—C7—C8	179.4 (3)	N1—C9—C10—F1	−59.5 (3)
C3—C2—C7—C6	0.5 (4)	N1—C9—C10—F2	−178.9 (2)

### Hydrogen-bond geometry (Å, °)

**Table:**

*D*—H···*A*	*D*—H	H···*A*	*D*···*A*	*D*—H···*A*
C6—H6···O2^i^	0.95	2.52	3.462 (3)	174
C4—H4···O1^ii^	0.95	2.60	3.229 (3)	124
C9—H9B···O2^iii^	0.99	2.54	3.311 (3)	135
C3—H3···F3^ii^	0.95	2.62	3.556 (3)	168
C3—H3···O1^ii^	0.95	2.66	3.247 (3)	120

Symmetry codes: (i) −*x*, −*y*+1, −*z*; (ii) −*x*+2, *y*−1/2, −*z*+1/2; (iii) *x*+1, *y*, *z*.
